# Psychometric Evaluation of the Decision Fatigue Scale among Korean Registered Nurses

**DOI:** 10.3390/healthcare12151524

**Published:** 2024-07-31

**Authors:** Yujin Hur, Ronald L. Hickman

**Affiliations:** 1 College of Nursing, Dongguk University WISE, Gyeongju 38066, Republic of Korea; 2 Frances Payne Bolton School of Nursing, Case Western Reserve University, Cleveland, OH 44106, USA

**Keywords:** decision making, nurses, factor analysis, reliability and validity

## Abstract

Nurses make decision for patients and the quality of nurses’ decision making can affect patient outcomes. For some reason, nurses are experiencing impaired decision making and it can negatively impact patient care. A valid and reliable instrument to assess decision fatigue may let people know about the concept and guide the development of new policies or interventions for Korean nurses’ decision fatigue. This study aimed to evaluate the psychometric properties of the Korean version of the decision fatigue scale. The design was a cross-sectional descriptive study and convenience sampling was used to recruit participants. A total of 247 nurses from across South Korea participated in an online survey. The survey consisted of demographic questionnaires, decision fatigue scale, nursing practice environment scale, and compassion fatigue scale. It was validated through confirmatory factor analysis that the Korean version of the decision fatigue scale was a single factor with the same structure as the original scale. The Korean version of the decision fatigue scale showed significant correlations with compassion fatigue, and the scale showed appropriate internal consistency. This study established well enough the psychometric characteristics of the Korean version of decision fatigue.

## 1. Introduction

Our daily lives are marked by making decisions, mostly for ourselves but often on behalf of others [1]. This is particularly true for healthcare professionals [2]. Decision making in nursing is a basic concept of systematic nursing practice that includes assessing, interpreting, evaluating, and managing each patient’s situation [3]. Registered nurses (RNs) make decisions continuously and the quality of nurses’ decision making can affect patient outcomes [4]. A nurse’s decision-making process is quite complex as it considers numerous competing factors, including the needs of patients and caregivers [5]. Yet, there are many cognitive and psychological factors, such as workload, cognitive load, impaired sleep, moral injury, and stress that can impair the ability of nurses to process information and make informed clinical decisions [6]. When nurses or individuals faced with decisions experience heightened states of psychological stress and other contributing factors that can influence the cognitive processing of information, these individuals are experiencing states of decision fatigue, which has been associated with impaired decision making in nurses and others faced with making decisions and negative decisional outcomes, such as decision regret [7]. Nurses’ decision fatigue is strongly correlated with traumatic stress and turnover intention, especially when the clinical environment is not appropriate [8]. Also, intensive care unit nurses reported that they experienced sleep disturbance due to decision-making regret [8]. These findings suggest that decision fatigue can hinder nurses from continuing their work. Additionally, nurses’ decision fatigue can lead to inappropriate decision making, causing negative impacts such as delays in hospitalization and patient prognosis [9,10,11,12].

To operationalize the concept of decision fatigue, the decision fatigue scale was developed by Hickman et al. [13]. This scale was based on the strength model of self-control [14], which describes ego depletion experienced through acts of self-control. The original version of the decision fatigue scale was developed as a nine-item scale that reflects the principal attributes of decision fatigue, which is posited as a unidimensional construct [13]. The decision fatigue scale was initially psychometrically evaluated in a cohort of surrogate decision makers for critically ill patients. After conducting exploratory and confirmatory factor analyses, the scale was reduced to nine items and was confirmed to have sufficient reliability and validity [13]. Subsequently, the decision fatigue scale has been used by researchers examining decision making in a broad ranges of cohorts, such as registered nurses, surrogate decision makers, and family caregivers [15,16,17], and even for different population groups, including clinical nurses, after psychometric evaluations [8]. Given the potential impact of decision fatigue on the clinical decision making of nurses, decision fatigue has been examined among Ame nurses as a factor that can contribute to reducing the quality of decision making and influence the well-being of nurses and their patients [8]. Nurses play especially important roles in decision-making processes about life-prolonging treatment and they want to be more involved in decisions [18]. 

Korea became an aged society in 2017 and it is expected to become a post-aged society by 2026 [19]. As one of the fastest aging countries, the Korean government is trying to prepare for the upcoming aging era. The “Act on hospice and palliative care and decisions on life-sustaining treatment for patients at the end of life” was enforced in Korea in February 2018 [20]. The purpose of this act is to respect self-determination of hospice care and life-sustaining treatment. In 2021, 57,511 patients in South Korea were affected by decisions to stop life-sustaining treatment, which was about 24.9% of total deaths in hospitals [21]. As we look at this change, we would expect that Korean nurses’ decision making roles would be expanded and also decision fatigue would increase accordingly. Furthermore, the nursing workplace environment has changed and nurses’ fatigue has accumulated due to the COVID-19 pandemic [22]. Workplace environments and nurses’ psychological status can affect nurses’ work performances and, further, it can influence nurses’ decision fatigue [17]. Specifically, compassion fatigue was heightened among nurses at risk of developing it during the pandemic and it particularly increases more and more among nurses experiencing patient suffering and death [23].

However, to our knowledge, decision fatigue has not been evaluated among Korean nurses. Relevant evidence indicates decision fatigue is relevant to Taiwanese patients and caregivers [16]. According to the need of the decision fatigue scale, this study validates the Korean version of the decision fatigue scale and figures out if there are any problems in using this scale in Korea. Therefore, the aim of this study was to evaluate the construct validity of the scale’s factor structure using confirmatory factor analysis and reliability of the Korean version of the decision fatigue scale.

## 2. Materials and Methods

### 2.1. Study Design

This was a cross-sectional descriptive study to evaluate the psychometrics of the K-DFS electronic recruitment methods that were used to enroll a convenience sample and surveys that were administered to participants electronically through an online survey website. 

### 2.2. Participants

The study participants were drawn from an online nurse forum, using a convenience sampling method. We set our goal of sample size as 250 participants, considering two conditions. First, the statistical power and precision of a confirmatory factor analysis (CFA) model required a ratio of at least 10 participants for every parameter [24]. Curran et al. [25] reported that when N was >200, the RMSEA was accurate for models with moderate misspecifications. To satisfy these two conditions, a minimum of 200 participants were needed. We set a goal to collect 250 participants to account for a dropout rate of 20%. Nurses who met the following criteria were eligible for this study: registered nurses who worked in the clinical setting during the time of the study, and registered nurses who were working with inpatient patients. Those ineligible for the study included pediatric nurses, registered nurses who did not involve direct patient care (e.g., nurse anesthetist, pharmaceutical nurse, infection control nurse, sterilization nurse), and registered nurses who did not work during the previous 24 h. 

### 2.3. Procedure

#### 2.3.1. Translation Procedures

We used the blind back translation technique to minimize translators’ bias [26]. This procedure consisted of three phases. First, the original English version of DFS was translated into Korean by a professional translator. Second, another professional translator translated the Korean versions back into English individually. Finally, the original scale developer and two bilingual nursing scholars checked for discrepancies between the content and meaning of the original version and translated version. After obtaining the result of the translation, the content validity was examined by six experts to determine whether the final adaptation where each translated tool was examined satisfied at least 0.78 of the item content validity index (I-CVI) and at least 0.90 of the scale content validity index/average (S-CVI/Ave) [27]. The expert group positively evaluated the translation tool for its semantic and conceptual equivalence and confirmed its overall cultural appropriateness.

#### 2.3.2. Data Collection Procedures

Data collection was administered through an online survey website and was conducted between 22 May 2022 and 17 April 2022. A total of 247 participants provided data. However, 36 participants were dropped because they did not fully complete the surveys. Our criterion for dropping participants was based on whether there was more than 50% missing data per case. Overall, it took participants on average 7–15 days to complete the surveys and no incentive was given to participants.

### 2.4. Measures

#### 2.4.1. Decision Fatigue Scale

The original decision fatigue scale was developed by Hickman et al. [13] in English to capture decision fatigue—a state of subjective low mental effort and behaviors that have been associated with irrational judgment. The decision fatigue scale was guided by the strength model of self-control and the relevant literature. It was intended to be a subjective measure covering various settings and decision-making types. Initially, the scale consisted of 13 items, and it was revised to a 10-item version based on experts’ recommendations. The psychometric evaluation resulted in the final version of the decision fatigue scale being modified to a nine-item single-factor solution. The decision fatigue scale was initially validated in a sample of surrogate decision makers of the critically ill and in a large cohort of registered nurses. The DFS is a self-report instrument for measuring respondents’ decision fatigue during the previous 24 h. This scale consists of 9 items, 1 dimension, and a 4-point Likert scale ranging from 0 (strongly disagree) to 3 (strongly agree). Higher total scores of each item mean a greater degree of decision fatigue. The scale evaluates psychometric properties at the developed point (adequate construct validity, discriminant validity, and reliability; Cronbach’s α was 0.87–0.90) [13] and, later, Pignatiello et al. [8] validated the scale for clinical nurses (adequate construct, convergent, discriminant validity, and reliability; Cronbach’s α was 0.95).

#### 2.4.2. Practice Environment Scale of the Nursing Environment

The practice environment scale of nursing work index (PES-NMI) was developed to measure the quality of the nurse practice environment [28]. This scale consisted of 31 items and 5 subdomains (nurse participation in hospital affairs, nursing foundations for quality of care, nurse manager ability, leadership, support of nurses, staffing and resource adequacy, and collegial nurse–physician relations). This scale is a 4-point scale ranging from 1 (strongly disagree) to 4 (strongly agree), and the higher mean score of each item means the better practice environment for nursing work. The original scale was validated with reliable construct validity and reliability (Cronbach’s α = 0.82). This scale was translated into Korean and validated with reliable reliability (Cronbach’s α was 0.93) and construct validity for the Korean nurses [29]. In this study, Cronbach’s α was 0.94.

#### 2.4.3. Compassionate Fatigue

ProQOL was used to measure compassion fatigue, professional burnout, and compassion satisfaction [30]. The professional quality of life (ProQOL) scale evolved from compassion fatigue self-test. Participants were asked to respond to statements indicating how often they have experienced each situation in the last 30 days using a Likert scale that ranged from 1 (never) to 5 (very often). The scale was validated with good construct validity, which has been translated into 28 languages and the α reliability for compassion fatigue was 0.81 in the original study, and Cronbach’s α was 0.85 in this study. 

#### 2.4.4. Demographic Characteristics

The demographic characteristics of participants measured in this study were age, gender, educational background, workplace characteristics, job experience, and turnover intention within six months.

### 2.5. Ethical Consideration

This study was conducted after being reviewed and approved by the Institutional Review Board of the relevant institute. A recruitment document was posted on the online nurses’ forum and participants voluntarily participated in the survey by clicking the link themselves. This study only included participants who agreed to participate in this study after reading the statement about research information, including the potential risks. Participants were able to discontinue their survey at any time. All collected data were stored and locked on an encrypted computer that only the researchers accessed. According to the Institutional Review Board policy, the collected data will be kept for three years and then completely deleted.

### 2.6. Data Analysis

The collected data were analyzed by using IBM SPSS 26.0 version and the Mplus 7.4 program in order to examine translated scale validity and reliability. A descriptive statistical analysis was performed by SPSS 26.0 version. The psychometric properties of the scale were evaluated by construct validity, convergent validity, discriminant validity, known group validity, and internal consistency.

#### 2.6.1. Confirmatory Factor Analysis

The construct validity was examined via only confirmatory factory analysis because this scale already confirmed factor structures [31]. CFA was sufficient as the tool was developed based on a theoretical framework, the factor structure was reported in the original tool, and the contextual meaning was validated during the translation procedure. CFA was performed by Mplus 7.4 and model fit was examined using the chi-squared test, root mean-square error of approximation (RMSEA), comparative fit index (CFI), Tucker–Lewis index (TLI), and standardized root mean-square residual (SRMR). 

#### 2.6.2. Convergent and Discriminant Validity

The convergent and discriminant validity was examined using the Mplus 7.4 program. This involved comparing the practice nursing environment, compassion fatigue, and the practice location. In order to demonstrate the DFS’s convergent validity, we hypothesized that it would show a moderate relationship between compassion and decision fatigue. Compassion fatigue and decision fatigue share several similarities, such as emotional exhaustion from nursing performance and stress-related symptoms, which can both lead to decreased job performance [30]. Our previous study has suggested that decision fatigue has a weak relationship with the nursing environment [8]. Based on this, we expected the association between the nursing environment and decision fatigue to be relatively weak. Even though the nursing environment may be linked to mental wellness, previous research results led us to assume that, in reality, it would demonstrate a weak rather than a direct relationship. To gain a clearer understanding of this, we included a question about work location to test for discriminant validity.

#### 2.6.3. Known Group Validity

The validity of the known group was examined using an independent *t*-test analysis of intention to leave and intention to stay within six months. Based on prior researches suggesting a strong correlation between decision fatigue among nurses and intention to leave their jobs [7,8], we aimed to examine the validity of decision fatigue between two groups: those with intent to leave and those without intent to leave.

#### 2.6.4. Reliability

The reliability of the K-DFS was examined using the Mplus 7.4 program, specifically in terms of internal consistency using Cronbach’s α coefficient. 

## 3. Results

### 3.1. Participants Characteristics

A total of 211 participants were included in this study (Table 1). The mean age was 32.19 years (range 22–53 years), and the majority were female (91.5%). The majority of participants were baccalaureate-prepared nurses (75.8%). Over half of the participants worked in capital areas (Seoul and Gyeonggi), which is congruent with national estimates that about 50% of nurses work in capital areas [32]. About half of the participants worked at general hospitals. On average, the participants had worked for 87.94 months (SD = 54.94).

### 3.2. Confirmatory Factor Analysis

Based on the results of the original version of DFS [13], single-factor structure was the hypothesis when conducting CFA. The K-DFS was normally distributed (Table 2). Standardized factor loadings ranged between 0.581 and 0.738 (Table 2). To verify the good fitness of the model, we evaluated the χ^2^ test, RMSEA, CFI, TLI, and SRMR. The value χ^2^ was 64.998 (df = 27, *p* < 0.001), but the χ^2^ test tended to reject the null hypothesis more than necessary [33]. The value of RMSEA was 0.082, which was below the cutoff of 0.10, showing a reasonable fit [34]. The values of CFI and TLI were 0.951, and 0.934, respectively. These results showed a satisfactory level of goodness of fit [35]. The SRMR was 0.040 (below 0.08 shows an acceptable level of goodness of fit [35]).

### 3.3. Convergent and Discriminant Validity

We evaluated K-DFS’s association with other scales to provide evidence of convergent and discriminant validity, as shown in Table 3 [36]. We assumed DFS’s convergent validity would possess a moderate relationship with the level of compassion fatigue. The K-DFS was excellently correlated with the CF (r = 0.646, *p* < 0.001), indicating excellent convergent validity, which is defined as over 0.60 being excellent [37]. We assumed DFS’s discriminant validity would possess a weak relationship with the PES-NWI and the questionnaire participants’ practice location (capital areas or not). The K-DFS was weakly correlated with the PES-NWI (r = −0.388, *p* < 0.001) and weakly correlated with the practice location (ρ = −0.179, *p* = 0.009), which met the criterion for discriminant validity (|r| < 0.70; [38]).

### 3.4. Known Group Validity

The mean K-DFS scores of the intent to turnover group (n = 63) and the stay group (n = 148) were 13.43 ± 6.21 and 11.55 ± 4.70, respectively (Table 4). The differences between groups were significantly different (t = 2.155, *p* = 0.034, d_Cohen_ = 0.36, 95% CI [0.06; 0.66]). Specifically, the intent to turnover group’s decision fatigue was higher than the stay group’s.

### 3.5. Reliability of the Korean Version of the Decision Fatigue Scale

Cronbach’s α for the scale was 0.88, which showed that the internal consistency of the Korean version was reliable because it met the criterion for internal consistency reliability greater than 0.80 [39]. In Table 5, the item total correlations, squared multiple correlations, and Cronbach’s alpha if item deleted for each item are provided. Based on the results, there was no necessity to remove any items [39].

## 4. Discussion

Registered nurses are responsible for a variety of health and healthcare decisions that are meant to optimize the health and well-being of the recipients of their care. The effectiveness of their clinical decision making is substantially impacted by factors, such as psychological stress, workload, compassion fatigue, and general health status, that have substantial impact on depleting the finite resources of their ego and prefrontal cortex (the anatomical region of the brain principally responsible for rationale judgment [40,41]). Depleted states of their ego and alterations in prefrontal cortical functioning are two hallmark conditions that predispose nurses and all individuals to states of decision fatigue [42]. The purpose of this study was to explore the validity and reliability of the decision fatigue scale in a cohort of Korean registered nurses. The results of the study provide evidence that decision fatigue as a concept is subjectively experienced by Korean nurses and that the translated version of the decision fatigue was supported by evidence of validity and reliability among Korean nurses. 

Consistent with prior studies, the Korean version of the decision fatigue scale demonstrated sufficient construct validity. In the present study, we conducted a confirmatory factor analysis to explore the structural validity of the Korean decision fatigue scale. The results of confirmatory factor analysis that established the Korean decision fatigue scale as a nine-item unidimensional scale was consistent with prior studies that examined the structural validity of the English version of the scale. In both published studies that explored the structural validity of the English version of the decision fatigue scale in surrogate decision makers and registered nurses, the nine-item unidimensional structure of the scale was shown to be the most parsimonious, which was further supported by consistently sufficient goodness-of-fit indices across samples [8,13]. The translated Korean version of the decision fatigue scale demonstrated equivalency to the English version of the scale in terms of structural validity. 

After assessing the structural validity of the Korean decision fatigue scale, convergent and discriminant validity were examined. Convergent validity was examined by assessing the strength of the relationship between scores of the Korean decision fatigue scale and the ProQoL’s compassion fatigue subscale. In the present study, we found a moderate correlation between the Korean decision fatigue scale and ProQoL compassion fatigue subscale scores, which supported the presence of convergent validity. The presence of convergent validity aligned with the evolving evidence base linking compassion fatigue (a subjective state of helplessness or exhaustion resulting from an exposure to a noxious psychological stimulus, such as workload or moral injury) and decision fatigue among registered nurses caring for patients during the COVID-19 pandemic. 

The third aspect in our assessment of the Korean decision fatigue scale’s construct validity was the evaluation of discriminant validity. Prior research demonstrated that the subjective appraisal of the work environment was linked to compassion fatigue and, to some extent, can affect decision fatigue among registered nurses [43]. Prior to this study, there had only been a single study conducted that explored the relationship between decision fatigue and the work environment of registered nurses. Pignatiello et al. [8] found a relatively modest correlation between decision fatigue and nurses’ appraisal of their work environment. Similarly, we found a small association between scores of the Korean decision fatigue scale and our measure of the work environment, PES-NWI, which substantiated discriminant validity between the instruments. Using the same instrument to capture the nursing environment, Pignatiello et al. [8] found that there was a statistically significant modest correlation between PES-NWI and decision fatigue, which supported the small interrelatedness between a healthy nursing environment and decision fatigue. Thus, the present study further corroborates prior research and highlights the relatedness of the workplace environment and decision fatigue among nurses, which is not direct.

Furthermore, employing known group validity as a strategy to substantiate the validity of the measurement variable, we utilized prior research findings, indicating a strong correlation between K-DFS and intention to leave [7,8]. We validated the validity of the K-DFS by analyzing differences between groups with and without the intention to leave. Based on these results, it was reevaluated that the concept of decision fatigue strongly correlated with nurses’ turnover intention. In future research, this result will need to be confirmed by repeating this study, and it is expected that it can be used to establish a strategic basis for improving nurses’ intention to stay and patient care outcomes.

Due to the cross-sectional nature of the present study, we were only able to assess the internal consistency reliability of Korean decision fatigue. The nine-item Korean decision fatigue scale was found to have an internal consistency reliability coefficient of 0.88. According to Cronbach [39], an internal consistency reliability coefficient greater than 0.80 is considered very good and the scale does not possess a high level of item redundance. When compared with the published studies reporting the internal consistency reliability coefficients for the decision fatigue scale, the Korean decision fatigue scale’s internal consistency reliability coefficient falls squarely within the range of coefficients previously reported.

A critical function of registered nurses in Korea and worldwide is their ability to make effective clinical decisions. In the present study, we provide evidence of a valid and reliable instrument to capture the concept of decision fatigue, which can influence the quality of the clinical decision making of nurses and others contemplating a decision. Having a valid and reliable instrument that captures decision fatigue introduces a new psychological and behavioral target for scientists, administrators, and policy makers that can help to ensure the delivery of the highest quality and most effective nursing care to patients and their families. Additionally, with a valid and reliable measure of decision fatigue, there is a new opportunity to develop new interventions, such as nursing-specific decision support systems, to alleviate decision fatigue among Korean nurses and further optimize their care delivery.

Although the established validity and reliability indicators of the K-DFS showed good metric characteristics, this study had three main limitations. First, the cross-sectional research design did not afford an opportunity to assess the stability of instrument over time. Second, the external generalizability of the Korean decision fatigue scale was limited to Korean nurses who identified as female, baccalaureate-prepared, and working in urban clinical settings. Lastly, given the electronic survey methodology, we were unable to capture potential individual, clinical, or practice-based confounders that may have influenced the responses of our participants. Despite these limitations, we implemented a rigorous translation process and a conventional psychometric approach to confirm the validity and reliability of the Korean decision fatigue scale. We suggest that future research should explore Korean nurses’ decision fatigue comprehensively and evaluate the level of decision fatigue using this scale. By measuring decision fatigue, many follow-up studies will be able to identify various variables related to decision fatigue, which is expected to improve nurses’ decision making.

## 5. Conclusions

To our knowledge, this was the first study to introduce the decision fatigue concept in South Korea. In this study, the original scale was translated into Korean and provided evidence of a reliable and valid scale for South Korean nurses. We provided evidence that the Korean version of the decision fatigue scale possessed the same unidimensional factor structure as the original scale. Also, we figured out that the score of the decision fatigue scale was excellently correlated with compassion fatigue and weakly correlated with the nursing practice environment. To conclude, this study provides evidence verifying the acceptable range of validity and liability of the Korean version of the decision fatigue scale in this study. We propose using K-DFS to evaluate the current decision fatigue of Korean nurses and develop strategies to reduce the negative effect of decision making on nurses. The validated Korean version of DFS will be widely employed by a multitude of researchers, making a substantial contribution to the accumulation of nursing knowledge regarding this concept and driving forward the field of nursing.

## Figures and Tables

**Table 1:** Demographic characteristics of the participants (N = 211).

Characteristics	n or Mean ± SD	% or Range
Age	32.19 ± 5.06	22–53
Gender		
Female	193	91.5
Male	18	8.5
Education		
Associates	30	14.2
Baccalaureate	160	75.8
Master’s	20	9.5
Doctorate	1	0.5
Region of practice		
Seoul	60	28.4
Gyeonggi	65	30.8
Gangwon	3	1.4
Chungcheong	30	14.2
Jeolla	24	11.4
Gyeongsang	29	13.7
Size of facility		
Senior general	58	27.5
General	106	50.2
Local clinic	47	22.3
Job experience (months)	87.94 ± 54.94	1–324

**Table 2:** Item statistics and factor loadings for the Korean version of the decision fatigue scale (N = 211). * *p* < 0.001.

Scale Items	Mean	SD	Skewness	Kurtosis	Estimate	S.E.	Standardized Estimate
I cannot make decisions because I am too tired or stressed.	1.42	0.754	0.151	−0.269	1.000	0.000	0.716 *
2. Making decisions is difficult because I cannot concentrate.	1.22	0.724	0.405	0.179	0.919	0.096	0.685 *
3. It has been hard for me to take in new information and use it.	1.34	0.779	0.297	−0.218	1.062	0.107	0.735 *
4. I do not have enough confidence in my ability to make good decisions.	1.40	0.847	0.119	−0.567	1.107	0.118	0.705 *
5. It takes too much effort to make decisions.	1.63	0.843	−0.080	−0.585	0.909	0.114	0.581 *
6. Someone should make decisions for me.	1.22	0.874	0.456	−0.375	1.044	0.118	0.644 *
7. I cannot make up my mind about which option is best.	1.39	0.857	0.244	−0.537	1.174	0.120	0.738 *
8. I have made decision without thinking carefully about them.	1.18	0.788	0.502	0.062	0.995	0.109	0.681 *
9. My mood has made it difficult for me to make decisions.	1.31	0.802	0.281	−0.302	0.963	0.110	0.648 *

**Table 3:** Convergent and discriminant validity of the Korean version of the decision fatigue scale (N = 211).

Variables	Practice Environment		Compassion Fatigue		Practice Location	
	r	*p*-Value	r	*p*-Value	ρ	*p*-Value
Decision Fatigue	−0.388	<0.001	0.646	<0.001	−0.179	0.009

**Table 4:** Known group validity of the Korean version of the decision fatigue scale (N = 211).

Group	n	Mean ± SD	t	*p*-Value	*d* (95% CI)
Intent to turnover	63	13.43 ± 6.21	2.155	0.034	0.36 (0.06–0.66)
Intent to stay	148	11.55 ± 4.70			

Abbreviations: SD—standard deviation; *d*—Cohen’s d coefficient; 95% CI, 95% confidence interval.

**Table 5:** Reliability of the Korean version of the decision fatigue scale.

Scale Items	Item Total Correlations	Squared Multiple Correlation	Cronbach’s Alpha If Item Deleted
1	0.67	0.52	0.87
2	0.65	0.50	0.87
3	0.68	0.50	0.87
4	0.65	0.48	0.87
5	0.55	0.32	0.88
6	0.60	0.39	0.87
7	0.68	0.52	0.87
8	0.63	0.43	0.87
9	0.61	0.38	0.87

## Data Availability

Data are available upon request from the corresponding author.
